# Protein-Enhanced Photoelectrochemical Sensor for Antioxidant Detection in Biodiesel Using Zinc Vanadate and Sulfur-Doped Graphitic Carbon Nitride

**DOI:** 10.1021/acsomega.5c02519

**Published:** 2025-06-20

**Authors:** Chirlene Nascimento Botelho, Jefferson Santos Oliveira, Adeilton Pereira Maciel, Gilvan Pereira de Figueredo, Flávio Santos Damos, Rita de Cássia Silva Luz

**Affiliations:** † Laboratory of Sensors, Devices and Analytical Methods, 37892 Federal University of Maranhão, 65080-805 São Luís, MA, Brazil; ‡ Postgraduate Program in Chemistry Associative UFMA-IFMA, Federal Institute of Education, 74353 Science and Technology of Maranhão, 65030-005 São Luís, MA, Brazil; § Fuel, Catalysis, and Environment Laboratory, Federal University of Maranhão, 65030-005 São Luís, MA, Brazil

## Abstract

Biodiesel stability is vital for performance, storage, and environmental benefits. However, oxidation and degradation can reduce its quality, leading to engine issues and reduced efficiency. Antioxidants are crucial to prevent these problems, but their detection remains a challenge. This study introduces a photoelectrochemical (PEC) sensor for detecting the 2-(1,1-dimethylethyl)-1,4-benzenediol (DBD) antioxidant in biodiesel. The sensor, constructed with FTO electrodes modified with sulfur-doped graphitic carbon nitride (g-C_3_N_4_–S), zinc vanadate (Zn_3_V_2_O_8_), and the protein cytochrome C (Cyt-c), showed a significantly higher PEC response compared to individual materials. Cyt-c enhances the photocurrent generation by reducing the recombination of photogenerated charges on the PEC platform surface. Photocurrent measurements were performed using amperometry with a 36 W white LED lamp as the irradiation source. Under optimized conditions, the sensor demonstrated two linear detection ranges: 0.6 to 160 and 160 to 2000 μmol L^–1^. The sensor was successfully applied to biodiesel samples, achieving recovery values between 98.99 and 105.26%, highlighting its accuracy and reliability for practical applications. This innovative approach addresses the challenges of detecting antioxidants in biodiesel, offering a sophisticated yet highly efficient solution to ensure fuel quality.

## Graphical abstract

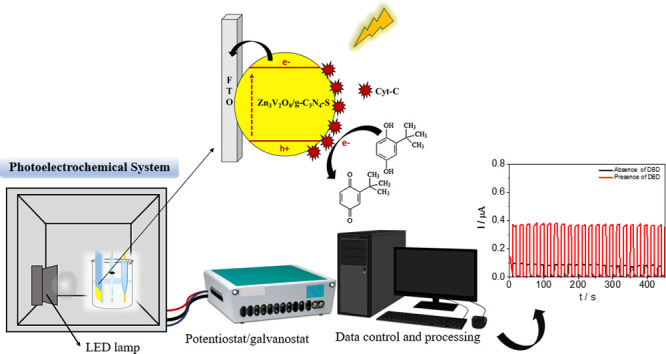

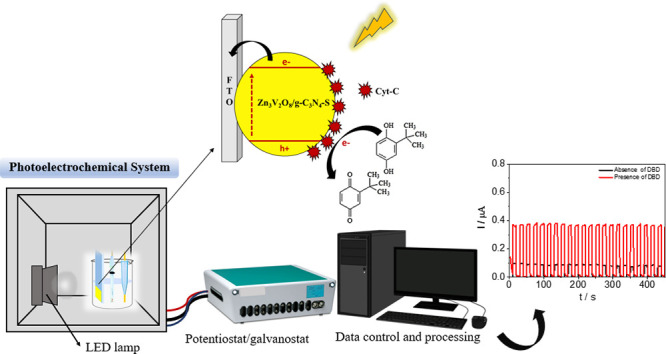

## Introduction

Biodiesel is a biofuel that plays a significant role in the economic and social development of nations. It is primarily produced through the transesterification of oils and fats, a process in which they react with an alcohol in the presence of a catalyst.^1^ This reaction yields long-chain fatty acid mono- and diesters, which can be either saturated or unsaturated.^1,2^ To ensure the high performance and stability of biodiesel, it is essential to minimize its exposure to air, as environmental factors such as heat, light, and other elements can accelerate the oxidation of its components, including aldehydes, ketones, and carboxylic acids, ultimately degrading its quality.^3^ The oxidation of biodiesel leads to the formation of peroxides and/or other compounds that mainly affect combustion efficiency, leading to corrosion in components such as the engine.^4^

To improve the performance of biodiesel, numerous antioxidants have been studied for their capacity to inhibit oxidation reactions.^5^ Among the most widely used synthetic antioxidants in edible oils and processed foods is 2-(1,1-Dimethylethyl)-1,4-benzenediol (DBD). Its primary role is to prevent oxidative degradation in these products.^6^ As a result, incorporating DBD into biodiesel plays a crucial role in enhancing the stability and longevity of this biofuel.^7^

A variety of methods for detecting antioxidants in oil or biodiesel samples have been reported in the literature. These include techniques such as batch injection analysis (BIA) coupled with pulsed amperometry,^8^ reverse-phase high-performance liquid chromatography (RP-HPLC),^9^ gas chromatography,^10^ electrochemical methods,^11–13^ calorimetric methods,^14^ and photoelectrochemical methods.^15^

Although methods such as HPLC and gas chromatography can accurately detect DBD,^16,17^ these techniques have some limitations. They often require large amounts of toxic and volatile organic solvents (e.g., methanol, isopropanol, *n*-hexane, and ethyl acetate).^17^ Additionally, they are sometimes unsuitable for analyzing fats and oils with high peroxide values due to interfering substances.^18^ Furthermore, gas chromatography-based methods sometimes face detection interference when nitrogen is used as the carrier gas.^18^ In addition, these methods exhibit high operational complexity, significant costs, and slow throughput analysis.^19^

In this context, photoelectrochemical (PEC) methods have emerged as a promising alternative for sample analysis, offering advantages such as ease of operation, cost-effectiveness, and rapid anaysis.^20,21^ The development of an effective PEC platform involves the integration of advanced analytical technology with semiconductor materials. These analyses are conducted under light illumination, where photoactive materials modified on the working electrodes generate a current signal, facilitated by electron transfer processes.^19,21^ As a result, photoelectrochemical sensors have become efficient tools for detecting various analytes, characterized by their structural simplicity, low production costs, potential for miniaturization, and the ability to achieve low detection limits with minimal sample volumes.^22^

Sensors utilizing working electrodes with immobilized redox proteins have seen significant advancements. Redox proteins-based detection technology has become a highly researched field, largely due to the sensitivity and selectivity of these systems.^23^ Building on this approach, we developed an innovative platform that combines photoactive materials sulfur-doped carbon nitride (g-C_3_N_4_–S), zinc vanadate (Zn_3_V_2_O_8_), and the redox protein cytochrome C (Cyt-c). These materials were integrated into a composite deposited on a fluorine-doped tin oxide (FTO) electrode.

g-C_3_N_4_, an n-type semiconductor, is valued for its chemical and thermal stability, as well as its moderate bandgap of 2.7 eV. However, its efficiency is often limited by the rapid recombination of electron–hole pairs. Sulfur doping modifies the positions of the valence and conduction bands, enhancing the electronic structure and improving charge mobility,^24^ like the effects observed in other carbon nitride systems doped with other nonmetals.^25^

Another notable photoactive material is Zn_3_V_2_O_8_, recognized for its photocatalytic properties,^26^ hydrogen storage capabilities, and energy storage potential.^27,28^ This compound is particularly significant due to its unique electronic properties and structural configuration, which consists of a combination of tetrahedra and octahedra, imparting distinctive characteristics for interactions with ionic and electronic species.^28^ On the other hand, the Cyt-c protein has been widely utilized in applications such as biomedicine,^29^ biotechnology,^30^ and electrochemistry,^31^ particularly in the development of biosensors for detecting various molecules.

In this study, zinc vanadate and sulfur-doped carbon nitride—cost-effective and easily synthesized materials—along with the enzyme Cyt-c, a key mediator in the charge transfer process, were employed for the determination of DBD. This combination offers several advantages, including rapid response, selectivity, and portability, making it a highly efficient and competitive alternative for PEC applications compared to other analytical methodologies.

In this context, the proposed sensor offers a simple and rapid detection under light irradiation, using minimal amounts of reagents, requiring only microliters of reagents and sample, while enabling the detection of low concentrations of DBD.

## Experimental Section

### Materials and Reagents

All reagents used were of analytical grade and did not undergo prior purification steps. The following chemicals were obtained from Isofar–Brazil: disodium phosphate (Na_2_HPO_4_), sodium hydroxide (NaOH), boric acid (H_3_BO_3_), ascorbic acid (C_6_H_8_O_6_, referred to as AA), phosphoric acid (H_3_PO_4_), monosodium phosphate (NaH_2_PO_4_), acetic acid (CH_3_COOH), zinc nitrate hexahydrate (Zn­(NO_3_)_2_·6H_2_O), and sodium sulfate (Na_2_SO_4_). Ammonium vanadate (NH_4_VO_3_), thiourea (CH_4_N_2_S), and cytochrome C (Cyt-c) were purchased from Sigma-Aldrich. Citric acid was sourced from LabSynth–Brazil. All solutions were purified in an OS100LXE system from GEHAKA Company.

### Synthesis of g-C_3_N_4_–S and Zn_3_V_2_O_8_

The sulfur-doped graphitic carbon nitride (g-C_3_N_4_–S) was synthesized through high-temperature heating. Initially, 5.0 g of thiourea were weighed into a crucible and placed in a muffle furnace, maintaining a temperature of 550 °C for 2 h. The entire procedure was carried out as described by Mohammad and co-workers.^32^

For the synthesis of zinc vanadate (Zn_3_V_2_O_8_), a liquid-phase precipitation procedure was adapted from Zhang and collaborators.^33^ Briefly, 0.3570 g of zinc nitrate hexahydrate (Zn­(NO_3_)_2_·6H_2_O) was weighed and dissolved in 8 mL of distilled water, forming solution 1. Meanwhile, 0.0931 g of ammonium vanadate (NH_4_VO_3_) was weighed and dissolved in 8 mL of distilled water, heated to 80 °C, forming solution 2. In the final step, solution 2 was slowly added to solution 1 under magnetic stirring for 2 h. The resulting sample was centrifuged, washed with ethanol and water, and dried at 60 °C in an oven. After this procedure, the product was calcined at 350 °C for 2 h, resulting in a yellowish product.

### Construction of the Photoelectrochemical Sensor

Initially, the FTO electrode was cleaned by immersion in ethanol and water. Then, after synthesizing the materials, 2 mg of g-C_3_N_4_–S and 2 mg of Zn_3_V_2_O_8_ were weighed and mixed with 15 μL of distilled water, forming a suspension. This suspension was sonicated for 20 min. After this procedure, 15 μL of the suspension were taken and added to the surface of the electrode. The modified electrode was left to dry at room temperature (25 °C) and then placed on a hot plate at 350 °C for 30 min. In the next step, a Cyt-c solution was prepared at a concentration of 10 mg mL^–1^, 10 μL of this solution was added to the FTO surface and allowed to dry for 30 min at room temperature (25 °C). Finally, a 1% Nafion solution was prepared, and a 10 μL aliquot was added to the surface of the electrode, resulting in the formation of the Cyt-c/Zn_3_V_2_O_8_/g-C_3_N_4_–S/FTO photoelectrochemical sensor.

### Structural and Morphological Characterization of the Materials by X-ray Diffraction (XRD), Fourier Transform Infrared Spectroscopy, Raman Spectroscopy and Scanning Electron Microscopy (SEM)

For X-ray diffraction measurements, a SHIMADZU XRD-6100 diffractometer with a Cu Kα radiation source (λ = 1.5406 Å) was used. The diffractometer operated at 30 kV and 30 mA, with an angular scan range (2θ) from 10° to 100°, a scan speed of 2°/min, and a step size of 0.02°.

Fourier Transform Infrared Spectroscopy (FTIR) Measurements are obtained with a Shimadzu IR- Prestige 21 coupled with ATR module. Measures were taken in the spectral range between 4000 and 400 cm^–1^. The Raman spectroscopy was performed using a micro-Raman system (Witec) equipped with a Nikon objective lens. An Argon ion laser operating at a wavelength of 514 nm was employed as the excitation source. To determine the morphological characteristics of the materials that compose the platform, Scanning Electron Microscopy (SEM) analyses were performed. SEM images were obtained using an EVO HD microscope (Zeiss, Jena, Germany).

### Electrochemical/Photoelectrochemical Measurements

Photoelectrochemical measurements were performed using an Autolab PGSTAT 128N potentiostat/galvanostat, controlled by Nova 2.1 software. A conventional three-electrode cell was employed, with fluorine-doped tin oxide (FTO) as the working electrode, a gold wire as the auxiliary electrode, and an Ag/AgCl electrode_(KClsat)_ as the reference electrode. A 36 W visible white light LED was used as the radiation source, housed in a custom-made box. All experiments were carried out with a fixed distance of 5 cm between the LED and the PEC cell, ensuring constant irradiance. Electrochemical impedance spectroscopy (EIS) measurements were performed in 0.1 mol L^–1^ Na_2_SO_4_. The measurements were performed in the presence and absence of white light from a LED lamp, covering a frequency range from 10^5^ Hz to 0.1 Hz. The impedance parameters were managed using the DropView 8400 software from Metrohm-DropSens.

### Application of the PEC Sensor in Biodiesel Samples

The preparation procedure of the biodiesel involved adding vegetable oil into a round-bottom flask, heated to 60 °C and subjected to mechanical agitation at 2000 rpm. A solution of KOH and methanol was then added, keeping the mixture agitated and heated for 45 min. After this process, the product was separated by decantation, and finally, the obtained biodiesel was washed and dried in an oven at 110 °C.

Subsequently, the Cyt-c/Zn_3_V_2_O_8_/g-C_3_N_4_–S/FTO PEC sensor was employed to determine DBD in biodiesel samples. The samples were prepared following the procedure described by Monteiro et al. Briefly, 1.0 g of biodiesel was weighed and diluted in 100.0 mL of concentrated ethanol. DBD was then spiked into the samples at two distinct concentrations (100 and 250 μmol L^–1^). A 10 μL aliquot of each sample was further diluted in 5.0 mL of 0.1 mol L^–1^ PBS (pH 6.0). The resulting solutions were analyzed using the external calibration method.

## Results and Discussion

### Characterization by X-ray Diffraction, Fourier Transform Infrared Spectroscopy, Raman Spectroscopy and Scanning Electron Microscopy of Zn_3_V_2_O_8_, g-C_3_N_4_–S, and Zn_3_V_2_O_8_/g-C_3_N_4_–S Materials

Figure 1 shows the zinc vanadate sample displayed the orthorhombic phase of Zn_3_V_2_O_8_, corresponding to the space group *Cmca* (ICSD 22943), as indicated by the diffraction peaks (2θ) at approximately 35°, 43°, and 63°. The phase identification was further validated through Rietveld refinement (Figure 1A). Similarly, the graphitic carbon nitride sample revealed its characteristic orthorhombic phase (Figure 1B), with a peak at around 13° attributed to the layered arrangement of tri-s-triazine units, assigned to the (100) plane, and a peak at 27° associated with the stacking of conjugated aromatic layers.^34^ These features align with the space group P21212 (ICSD 194747), which was also confirmed via refinement. Furthermore, the absence of additional peaks in both samples suggests a high degree of phase purity, with no detectable impurities or secondary phases. The refinement parameters are detailed in Table 1.

**Figure 1:**
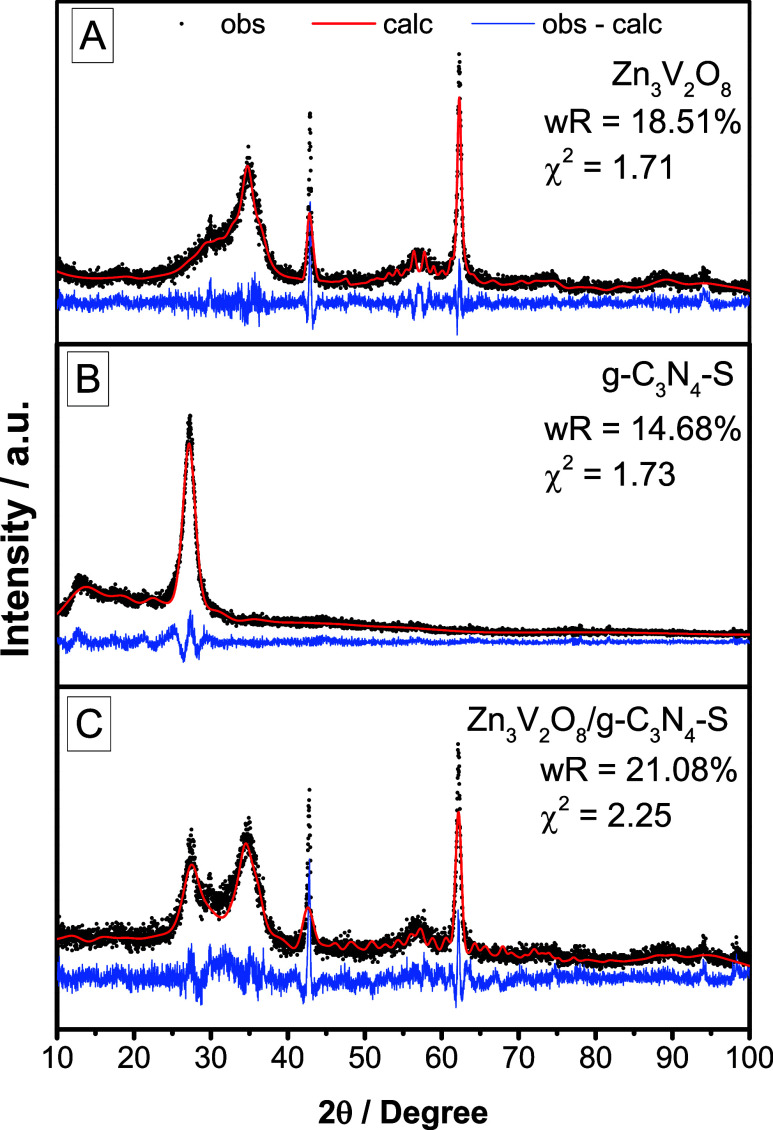
XRD patterns of (a) Zn_3_V_2_O_8_, (b) g-C_3_N_4_–S, (c) Zn_3_V_2_O_8_/g-C_3_N_4_–S with respective Rietveld refinement.

**Table 1:** Lattice Parameters of Zinc Vanadate (Zn_3_V_2_O_8_), Sulfur Doped Graphitic Carbon Nitride (g-C_3_N_4_–S) and Their Composite

			lattice parameters (Å)			reliability parameters	
sample	phase	phase fraction (%)	*A*	*B*	*C*	w*R* (%)	*X*
Zn_3_V_2_O_8_	ICSD 22943	monophasic	6.12	11.52	8.09	18.51	1.71
g-C_3_N_4_–S	ICSD 194747	monophasic	15.17	12.77	6.56	14.68	1.73
Zn_3_V_2_O_8_/g-C_3_N_4_–S	ICSD 194747	66.40%	6.12	11.29	8.15	21.08	2.25
	ICSD 22943	33.60%	15.46	11.84	6.41		

For the composite sample (Figure 1C), as expected, the phases of zinc vanadate and sulfur-doped graphitic carbon nitride are observed with no additional phases, indicating the successful preparation of Zn_3_V_2_O_8_/g-C_3_N_4_–S. The mixture comprises 66.40% zinc vanadate phase and 33.60% graphitic carbon nitride phase.

FTIR spectra of g-C_3_N_4_–S, Zn_3_V_2_O_8_, and their composite are displayed in Figure 2a. Typical bands of graphitic carbon nitride are observed for g-C_3_N_4_–S. A broad, low intensity band registered between 3334 and 3019 cm^–1^ is assigned to N–H stretching.^35,36^ Its low intensity is typical for sulfur doped graphitic nitride carbon, because doping S atoms can replace NH_2_ groups.^37^ The band at 2980 can be assigned to O–H stretching. Intense bands between 1200–1600 cm^–1^ are attributed to aromatic C–N stretching.^35,36,38^ In addition, a high intense vibrational mode at 810 cm^–1^ corresponds to tris-S-triazine group, ensuring S doping.^36,39^

**Figure 2:**
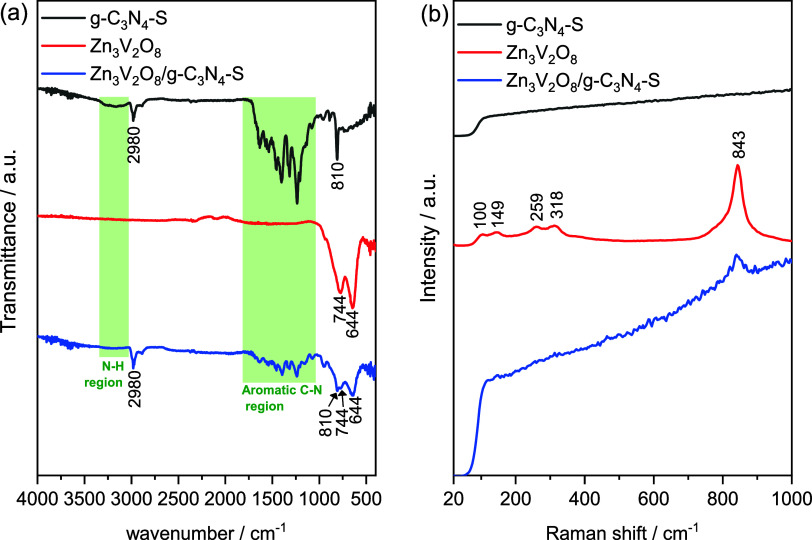
FTIR (a) and Raman (b) spectra for g-C_3_N_4_–S, Zn_3_V_2_O_8_, and Zn_3_V_2_O_8_/g-C_3_N_4_–S.

For zinc vanadate, the bands between 401 and 530 cm^–1^ that can be assigned to ZnO stretching, and other between 600 and 780 cm^–1^, characteristic of V–O–V vibration can be observed.^40,41^ The composite spectrum retains key bands from both precursors: the tris-S-triazine vibration (810 cm^–1^) and V–O–V modes (744 cm^–1^), alongside Zn–O and C–N signals, confirming the coexistence of both phases. This aligns with XRD results, validating the composite’s successful synthesis. Notably, minor peak broadening and reduced intensity in the composite suggest interfacial interactions between g-C_3_N_4_–S and Zn_3_V_2_O_8_.

Raman spectra of the g-C_3_N_4_–S, Zn_3_V_2_O_8_, and their composite in the region of 20–1000 cm^–1^ are presented in Figure 2b. Distinct bands are observed at 100, 149, 259, 318, and 843 cm^–1^. The low-frequency bands at 100, 149, and 259 cm^–1^ are attributed to V–O bending modes,^42–44^ while the 318 cm^–1^ likely corresponds to overlapping contributions from Zn–O vibrations and asymmetric O–V–O bending modes.^42,44^ The prominent high-frequency band at 843 cm^–1^ is assigned to the V–O stretching mode,^42–44^ consistent with the structural framework of zinc vanadate (Zn_3_V_2_O_8_) as confirmed by XRD analysis. In contrast, no discernible Raman bands were detected for g-C_3_N_4_–S within this spectral range. These results collectively corroborate the formation of the composite material with the presence of Zn_3_V_2_O_8_ and g-C_3_N_4_–S.

Figure 3a presents the scanning electron microscopy (SEM) images of the synthesized materials: zinc vanadate (Zn_3_V_2_O_8_), sulfur-doped graphitic carbon nitride (g-C_3_N_4_–S), and the composite material (Zn_3_V_2_O_8_/g-C_3_N_4_–S). The SEM image of Zn_3_V_2_O_8_ reveals a hierarchical microsphere morphology composed of intercrossing nanoflakes, resembling layers of flakes.^45,46^ Figure 3b shows g-C_3_N_4_–S, which exhibits a large number of irregular blocky agglomerates.^32,47^ This structure of g-C_3_N_4_–S is favorable for anchoring the Zn_3_V_2_O_8_ microspheres, as observed in Figure 3c, which depicts the composite material Zn_3_V_2_O_8_/g-C_3_N_4_–S, confirming the successful combination of these materials.

**Figure 3:**
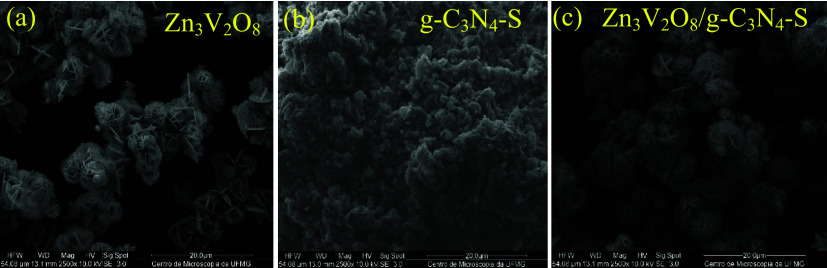
SEM images of: (a) Zinc Vanadate (Zn_3_V_2_O_8_); (b) Sulfur-doped Graphitic Carbon Nitride (g-C_3_N_4_–S); (c) Composite material Zn_3_V_2_O_8_/g-C_3_N_4_–S.

### Electrochemical Characterization of the PEC Sensor

Figure 4a shows the photoelectrochemical measurements of the proposed platform in the absence (red amperogram) and presence (pink amperogram) of the Cyt-c protein, as well as in the absence and presence of DBD. It is observed that with the presence of Cyt-c, there is a considerable increase in photocurrent compared to the platform without Cyt-c. This suggests that the protein further reduces the recombination of photogenerated charges on the PEC platform’s surface, resulting in a higher photocurrent for the system.

**Figure 4:**
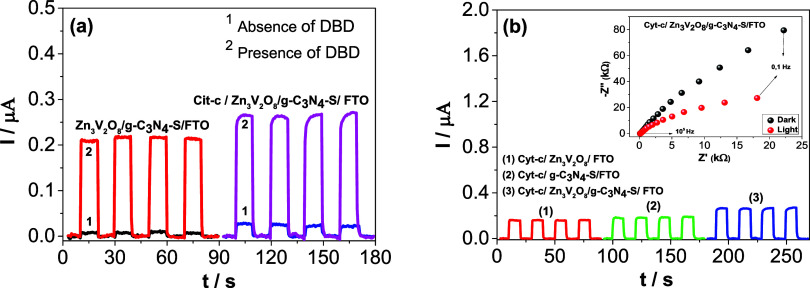
(a) Amperograms obtained for the Zn_3_V_2_O_8_/g-C_3_N_4_–S/FTO platform in the absence (red amperogram) and presence (pink amperogram) of Cyt-c, as well as in the presence and absence of DBD. (b) Amperograms obtained for: (1) Cyt-c/Zn_3_V_2_O_8_/FTO (red amperogram); (2) Cyt-c/g-C_3_N_4_–S/FTO (green amperogram); (3) Cyt-c/Zn_3_V_2_O_8_/g-C_3_N_4_–S/FTO (blue amperogram). Experiments were performed in PBS at pH 7.0, with the presence of DBD (1 mmol L^–1^). *E*_appl._ = 0.3 V vs Ag/AgCl _(KClsat)_. Inset of (b): Nyquist plots for the Cyt-c/Zn_3_V_2_O_8_/g-C_3_N_4_–S/FTO platform with and without visible LED light exposure.

Concurrently, Figure 4b presents the amperometric measurements for the immobilized Cyt-c/Zn_3_V_2_O_8_ (red amperogram), Cyt-c/g-C_3_N_4_–S (green amperogram), and Cyt-c/Zn_3_V_2_O_8_/g-C_3_N_4_–S (blue amperogram) on the FTO electrode. The experiments were performed in PBS at pH 7.0, containing 1 mmol L^–1^ DBD, with an applied potential of *E*_appl_. = 0.3 V vs Ag/AgCl _(KClsat)_. The complete Cyt-c/Zn_3_V_2_O_8_/g-C_3_N_4_–S/FTO platform exhibited a significantly higher photocurrent compared to the individual platforms Cyt-c/Zn_3_V_2_O_8_/FTO and Cyt-c/g-C_3_N_4_–S/FTO, suggesting that a synergy occurs between the materials in the complete platform. Zn_3_V_2_O_8_ has excellent charge transfer properties, g-C_3_N_4_–S may enhance light absorption, and the Cyt-c protein acts as a facilitator in electron transfer. Therefore, the combination of these materials promoted efficient photosensitization in the system.

The inset of Figure 4b refers to the Nyquist plot for the Cyt-c/Zn_3_V_2_O_8_/g-C_3_N_4_–S/FTO platform. The experiments were carried out in 0.1 mol L^–1^ Na_2_SO_4_ in the presence and absence of white LED light. The results show lower charge transfer resistance when the platform is illuminated, resulting in efficient generation of charge carriers (*e* ^–^/h^+^).

Figure 5a shows the amperometric measurements for the PEC sensor at different pH values, ranging from 5 to 8. The inset in Figure 5a illustrates the variation of photocurrent at different pH values. All measurements were obtained in 0.1 mol L^–1^ PBS at pH 7.0, with *E*_appl._ = 0.3 V, containing 1 mmol L^–1^ DBD. It was observed that photocurrent values increased from pH 5 to 6, and beyond this pH, the photocurrent values decreased and remained nearly constant. This result suggests that the photoactive material and the redox properties of DBD were favorable for the molecule to respond well to light, generating a significant photocurrent at pH 6.0, resulting in a more efficient interaction with the sensor components, evidencing the suitability of this pH for sensitive detection of the antioxidant.

**Figure 5:**
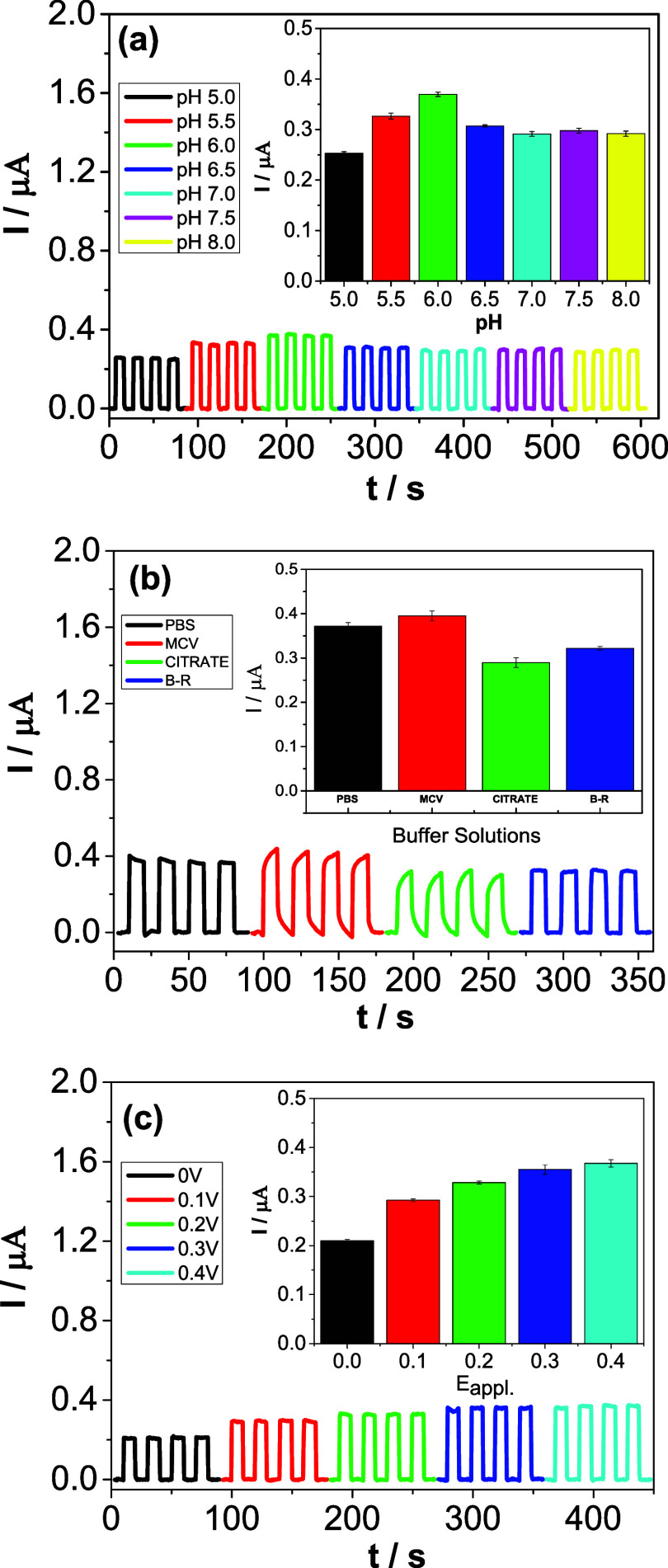
(a) Amperograms obtained for the Cyt-c/Zn_3_V_2_O_8_/g-C_3_N_4_–S/FTO platform at different pH values in the presence of 1 mmol L^–1^ DBD. *E*_appl_ = 0.3 V vs Ag/AgCl _(KClsat)_. Inset of ([Fig fig5]a): Photocurrent as a function of pH. (b) Amperograms obtained for different types of buffer solutions (0.1 mol L^–1^, pH 6.0) containing 1 mmol L^–1^ DBD. Inset of (b): Photocurrent vs buffer solution type. (c) Amperograms for different applied potential values (0–0.4 V vs Ag/AgCl _(KClsat)_). The study was conducted in 0.1 mol L^–1^ PBS, pH 6.0, containing 1 mmol L^–1^ DBD. Inset of (c): Photocurrent vs applied potential.

Subsequently, Figure 5b presents the amperograms obtained in different buffer solutions (Britton-Robinson–BR; Phosphate–PBS; McIlvaine–MCV; Citrate). The inset in Figure 5b shows the photocurrent graph for each buffer solution. The measurements were obtained in 0.1 mol L^–1^ of each buffer, at pH 6.0, with *E*_appl._ = 0.3 V vs Ag/AgCl _(KClsat)_, containing 1 mmol L^–1^ DBD. The highest photocurrent was obtained for the MCV buffer; however, compared to the PBS, no significant difference in photocurrent was observed. To ensure the stability of the photocurrents and the repeatability of the measurements, the PBS was chosen for subsequent measurements. With the experimental conditions for pH and buffer (pH 6.0 and PBS) fixed, the applied potential (*E*_appl_) study was conducted. Figure 5c shows the amperometric measurements obtained at different potentials (0–0.4 V vs Ag/AgCl _(KClsat)_). The inset in Figure 5c shows the variation in photocurrent for each potential. The results indicate a proportional increase in photocurrent with increasing potential values. In this context, aiming for charge transfer efficiency and system sensitivity, a potential of 0.3 V vs Ag/AgCl_(KClsat)_ was applied to the working electrode.

The construction of the analytical calibration curve using the PEC sensor for the detection of the DBD molecule is illustrated in Figure 6. Specifically, Figure 6a displays the amperometric responses obtained from the Cyt-c/Zn_3_V_2_O_8_/g-C_3_N_4_–S/FTO platform across varying concentrations of DBD. As the concentration of the analyte increases, the photocurrent also increases until it reaches a plateau. Figure 6b illustrates the photocurrent intensity as a function of the analyte concentration, with two distinct linear ranges: the first from 0.6 to 160 μmol L^–1^, and the second from 160 to 2000 μmol L^–1^. The linear regression equations were Δ*I*/μA = 0.016 + 0.0011 ([DBD] μmol L^–1^) with a correlation coefficient of 0.994, and Δ*I*/μA = 0.19 + 5.65 × 10^–5^ ([DBD] μmol L^–1^), with a correlation coefficient of 0.990. The detection limit (LD) was determined considering the Signal-to-Noise ratio (S/N = 3), resulting in a value of 0.002 μmol L^–1^. The PEC sensor detected DBD with high sensitivity (0.6 μM or 0.113 ppm), well below the industrial minimum requirement (50–100 ppm) for biodiesel oxidative stability.^48,49^ However, the presence of high amounts of foreing substances from biodiesel matrix may generate overlapping signals when very low concentrations of DBD are determined. These effects become negligible within the established linear range (0.6–2000 μmol L^–1^), which confirms the selectivity of the sensor to DBD detection. Subsequently, the analytical characteristics of the proposed sensor were compared with other studies reported in the literature, as shown in Table 2,^10–12,50–53^ demonstrating that the developed method exhibits comparable or superior performance to those described in the literature. Regarding the PEC platform, it is important to highlight that most platforms developed for the detection of molecules such as DBD use a xenon (Xe) lamp as the light source.^52^ This type of lamp is relatively expensive, making the system high-cost, whereas the proposed system employs a 36 W white LED lamp, which was purchased from a local store.

**Figure 6:**
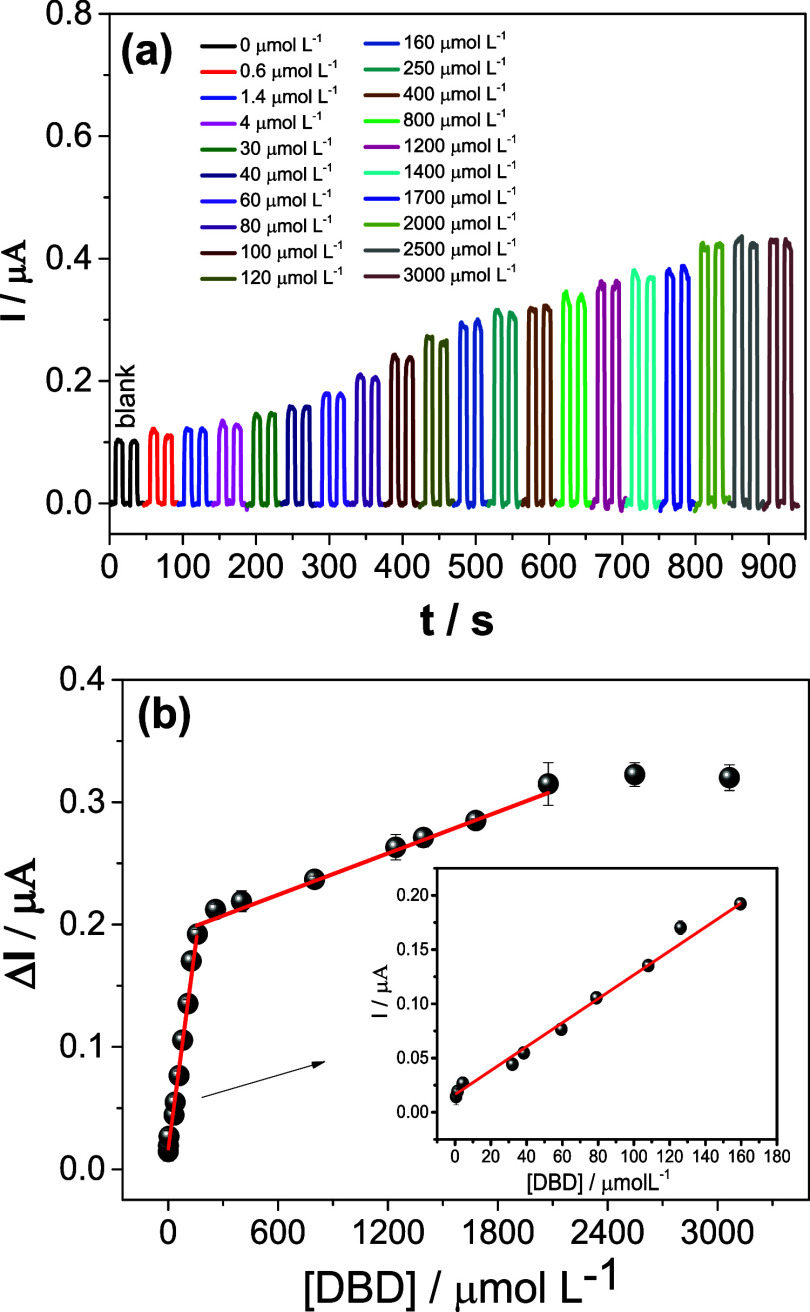
(a) Photocurrent responses obtained for the Cyt-c/Zn_3_V_2_O_8_/g-C_3_N_4_–S/FTO PEC sensor for the blank (black amperogram) and different DBD concentrations (0.6 to 3000 μmol L^–1^). (b) Analytical curve for DBD detection using the Cyt-c/Zn_3_V_2_O_8_/g-C_3_N_4_–S/FTO platform. Inset of (b): extension of the first concentration range. Measurements were performed in 0.1 mol L^–1^ PBS (pH 6.0), with *E*_appl_ = 0.3 V vs Ag/AgCl_(KCl sat)_.

**Table 2:** Comparison of Analytical Parameters for DBD Determination^a^

techniques	detection limit (μmol L ^–1^)	linear range (μmol L ^–1^)	refs
LSV	0.34	0.5–10	^10^
DPV	0.14	0.40–120	^11^
DPV	0.42	1.0–75.0	^12^
DPV	0.0067	0.025–100	^50^
LSV	0.48	1.2–16.85	^51^
PEC	0.526	1.00–500	^52^
DPV	0.001	0.12–62.2	^53^
PEC	0.002	0.6–160	this work
		160–2000	

a DPV: differential pulse voltammetry; LSV: linear sweep voltammetry; PEC: photoelectrochemical.

Additionally, a mechanism for the Cyt-c/Zn_3_V_2_O_8_/g-C_3_N_4_–S/FTO sensor in the detection of DBD using white LED light is proposed, as illustrated in Scheme 1. In this context, it is important to highlight the role of each component in the PEC detection platform. Graphitic carbon nitride (g-C_3_N_4_) exhibits exceptional photoelectrochemical properties; however, it suffers from drawbacks such as rapid electron–hole recombination, slow charge mobility, and low light absorption capacity.^54,55^ Sulfur doping helps overcome these limitations by narrowing the bandgap, enhancing visible light absorption, and creating more active sites, which results in improved charge carrier separation efficiency and overall material performance.^56^ Thus, g-C_3_N_4_–S acts as a photocatalyst under light irradiation.

**Scheme 1:**
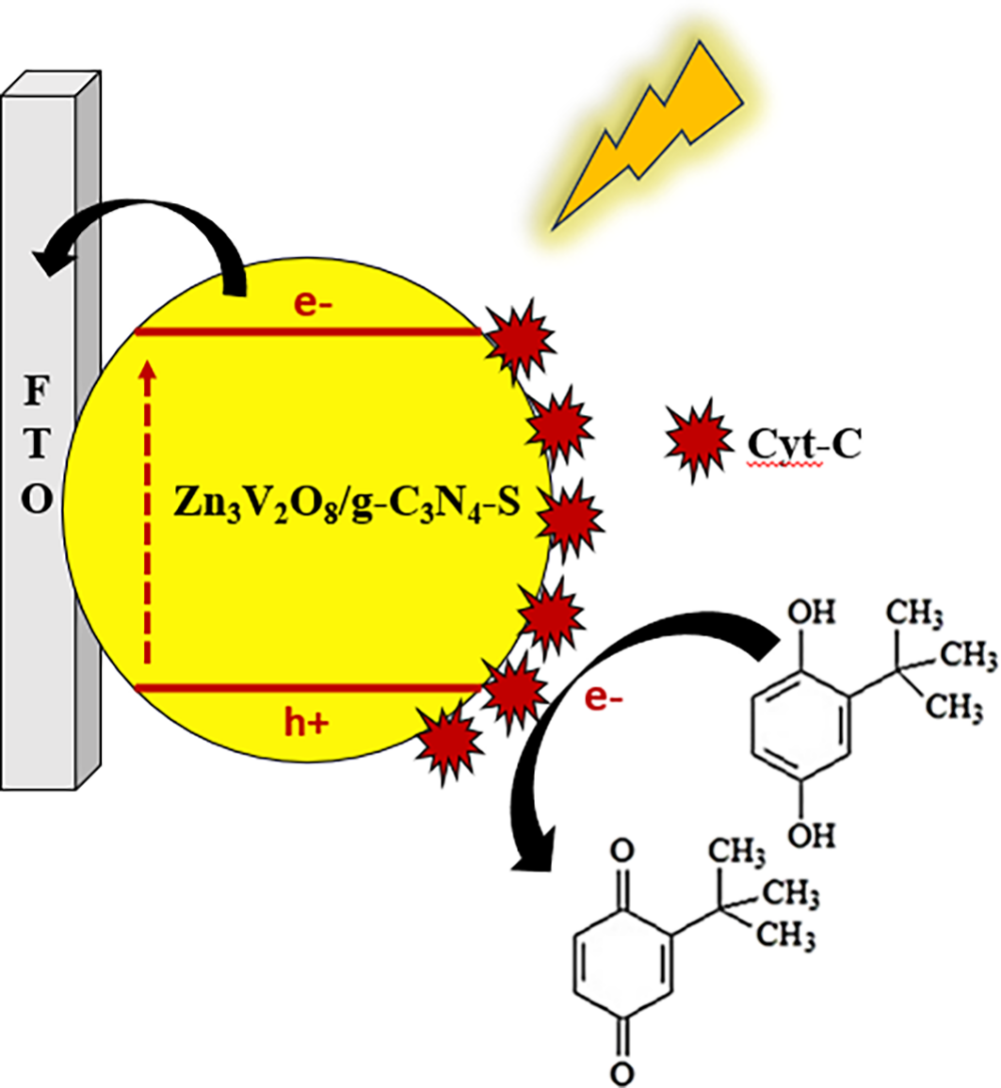
Proposed Mechanism for PEC Detection of DBD Using the Cyt-c/Zn_3_V_2_O_8_/g-C_3_N_4_–S/FTO Platform

Subsequently, zinc vanadate (Zn_3_V_2_O_8_), with its narrow bandgap, extends the absorption range in the solar spectrum and accelerates charge separation.^57,58^ Zn_3_V_2_O_8_ functions as an electron acceptor, promoting efficient separation of the photogenerated electron–hole pairs upon light incidence. Finally, cytochrome c (Cyt-c), a redox-active protein, plays a key role as an electron transfer facilitator and also contributes to enhancing the biocompatibility of the biosensor.^59^ In this sense, Cyt-c acts as a redox mediator, enabling rapid electron transfer within the platform.

Therefore, according to the illustration, the composite material strongly absorbs visible light, generating electrons in the valence band (VB) and the conduction band (CB). Subsequently, DBD acts as an electron donor, capturing the holes in the Zn_3_V_2_O_8_/g-C_3_N_4_–S composite. Simultaneously, the enzyme Cyt-c immobilized on the platform interacts with the composite material, facilitating the electron transfer process.

### Study of Measurement Repeatability, Reproducibility, and Selectivity

Figure 7 illustrates the evaluation of the repeatability, reproducibility, and selectivity of the Cyt-c/Zn_3_V_2_O_8_/g-C_3_N_4_–S/FTO PEC sensor for DBD detection. Figure 7a demonstrates the repeatability of the sensor measurements over a 500 s interval in the absence and presence of 1000 μmol L^–1^ DBD. The relative standard deviation (RSD) values of 3.78% (absence of DBD) and 1.34% (presence of DBD) indicate high accuracy and consistent performance of the sensor within a single day. This result confirms the robustness of the developed sensor under consistent operating conditions. Figure 7b presents the reproducibility of the sensor by comparing the amperometric measurements obtained on different days using five independently prepared electrodes in the presence of 1 mmol L^–1^ DBD. Similar results are observed, even with the natural variation of time, the RSD value of 3.77% demonstrates excellent reproducibility and reliability of the sensor in different experimental configurations, reinforcing its potential for practical applications in DBD detection. Finally, Figure 7c explores the selectivity of the sensor by evaluating potential interferents, including Sodium ions (Na^+^), Ascorbic acid (AA), Butylated hydroxyanisole (BHA), Calcium ions (Ca^2+^), Potassium ions (K^+^), and Butylated hydroxytoluene (BHT). The results show no significant change in photocurrent percentage in the presence of these interferents, indicating that the Cyt-c/Zn_3_V_2_O_8_/g-C_3_N_4_–S/FTO sensor exhibits excellent selectivity for DBD detection. This selectivity ensures that the sensor can accurately detect DBD in complex sample matrices without interference from other common substances. The presence of high amounts of foreing substances from biodiesel matrix may generate overlapping signals when very low concentrations of DBD are determined. These effects become negligible within the established linear range (0.6–2000 μmol L^–1^), which confirms the selectivity of the sensor to DBD detection. In summary, the developed Cyt-c/Zn_3_V_2_O_8_/g-C_3_N_4_–S/FTO PEC sensor demonstrates high precision, reproducibility, and selectivity for DBD detection, making it a reliable tool for the quality control and analysis of biofuels.

**Figure 7:**
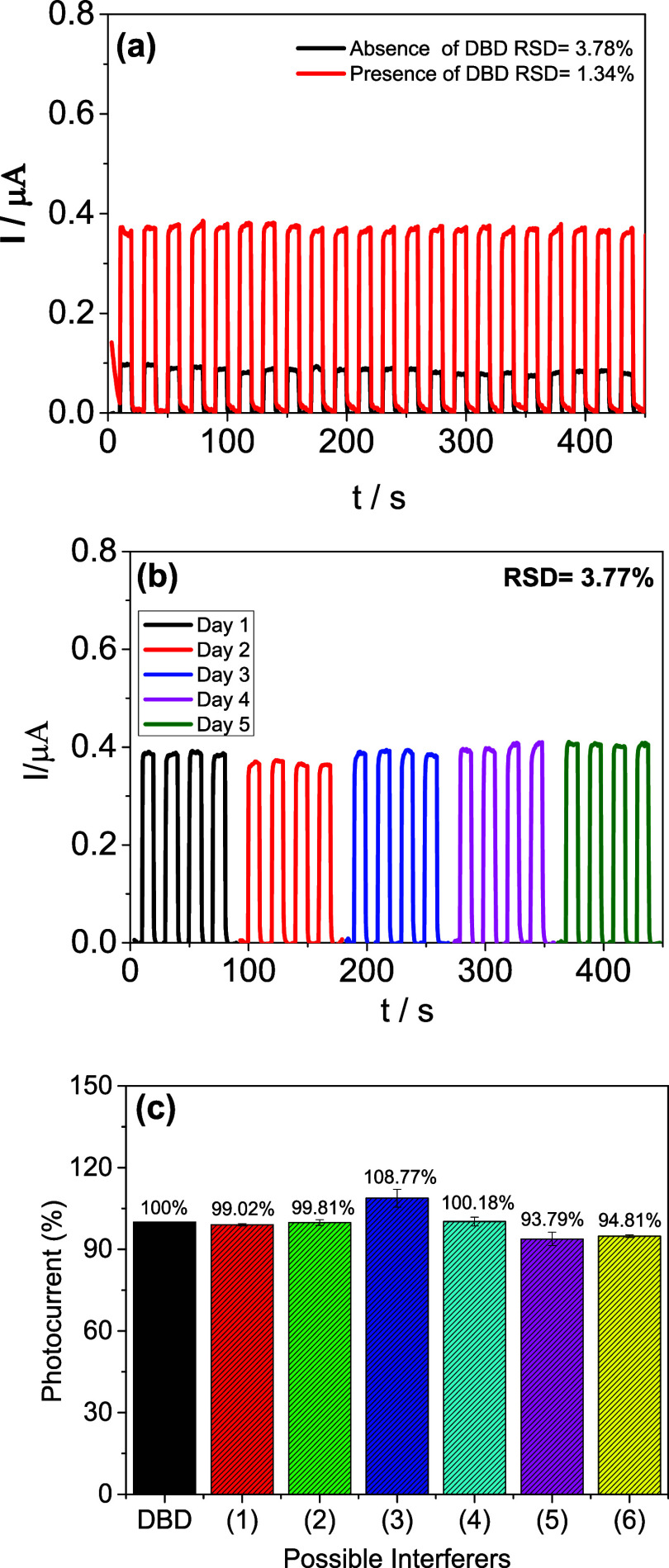
(a) Evaluation of the repeatability of measurements in 500 s for the Cyt-c/Zn_3_V_2_O_8_/g-C_3_N_4_–S/FTO PEC sensor in the absence and presence of DBD. (b) Amperograms obtained on different days for the proposed sensor in the presence of 1 mmol L^–1^ DBD. Studies carried out in PBS 0.1 mol L^–1^, pH 6.0, *E*_appl._ = 0.3 V, containing 1 mmol L^–1^ DBD. (c) Study of possible interferents for DBD, (1) Sodium ions (Na^+^); (2) Ascorbic acid (AA); (3) Butylated hydroxyanisole (BHA); (4) Calcium ions (Ca^2+^); (5) Potassium ions (K^+^); (6) Butylated hydroxytulene (BHT). Study carried out in PBS 0.1 mol L^–1^, pH 6.0, *E*_appl._ = 0.3 V, containing 100 μmol L^–1^ DBD and possible interferents.

### Application of the Photoelectrochemical Sensor in Biodiesel Samples

The accuracy and applicability of the developed Cyt-c/Zn_3_V_2_O_8_/g-C_3_N_4_–S/FTO platform were tested to determine DBD at different concentrations in biodiesel samples. The samples were spiked at two concentrations (100 and 250 μmol L^–1^), and the quantification of DBD in the spiked samples was determined using the external calibration method.

Table 3 presents the recovery values obtained, which ranged from 98.99 to 105.26%, accompanied by low standard deviations. These results indicate good accuracy and precision, demonstrating reliability in the methodology used, being effective for the detection of DBD in real biodiesel samples.

**Table 3:** Results of DBD Detection in Biodiesel Sample Using PEC Sensor Cyt-c/Zn_3_V_2_O_8_/g-C_3_N_4_–S/FTO

samples	[DBD] added (μmol L^–1^)	[DBD] found (μmol L^–1^)	recovery (%)
Babassu Biodiesel			
B1	100	104.93 (±0.0019)	104.93
B2	250	262.98 (±0.0018)	105.19
Soy Biodiesel			
S1	100	98.99 (±0.0031)	98.99
S2	250	263.17 (±0.0045)	105.26

### Influence of Environmental Factors on Sensor Performance

The accuracy of the sensor can be influenced by environmental factors such as temperature, humidity, and pressure, which may directly affect its performance. In this study, all preliminary tests were conducted under controlled laboratory conditions: room temperature (25 ± 1 °C), a mean relative humidity of about 50%, and standard atmospheric pressure (1 atm). The sensor demonstrated excellent stability under these conditions. However, for practical applications in diverse environments, additional studies are recommended to ensure reliable performance across varying scenarios

## Conclusions

This work describes the development of a PEC sensor for the detection of DBD in biodiesel samples. The system is based on a commercially available low-cost 36 W LED light combined with a homemade box to control light incidence. The sensor demonstrated an excellent response under light exposure, and the Cyt-c present on the platform reduces the recombination of photogenerated charges on the PEC platform’s surface, resulting in a high photocurrent for the system. Material characterization confirmed each material and its interaction, which, under the presence of visible LED light, led to a wide linear response range and a low detection limit. Therefore, the developed PEC sensor can be considered a promising proposal for determining DBD in biodiesel samples, showing recovery values ranging from 98.99 to 105.26%, as well as good selectivity, precision, and accuracy.
